# Exploring patterns of distributional justice in global climate change mitigation scenarios

**DOI:** 10.1038/s44168-026-00364-4

**Published:** 2026-03-31

**Authors:** Karl Scheifinger, Elina Brutschin, Kian Mintz-Woo, Caroline Zimm, Jarmo S. Kikstra, Joeri Rogelj, Piotr Żebrowski, Thomas Schinko, Shonali Pachauri, Benjamin K. Sovacool, Livia Fritz, Keywan Riahi

**Affiliations:** 1 Centre for Environmental Policy, Imperial College London, London, UK; 2 International Institute for Applied Systems Analysis, Laxenburg, Austria; 3 Webster Vienna Private University, Vienna, Austria; 4 Philosophy and Sustainability Institute, University College Cork, Cork, Ireland; 5 Centre for Environmental Policy and Grantham Institute—Climate Change and Environment, Imperial College London, London, UK; 6 Department of Business Development and Technology, Aarhus University, Aarhus, Denmark; 7 Department of Earth and Environment, Boston University, Boston, MA USA; 8 Department of Geography and Environment and Institute of Environmental Studies, University of Geneva, Geneva, Switzerland

**Keywords:** Climate sciences, Environmental social sciences

## Abstract

Collective climate action hinges on the distribution of benefits and burdens of climate change mitigation. Yet assumptions relevant to distributional justice are frequently made only implicitly in climate change mitigation scenarios. Here, we introduce the patterns of the distributional justice framework that operationalize philosophical justice theories as quantitative requirements for scenario trajectories. We then apply this framework to the IPCC AR6 scenario database to assess the distributional implications of global climate change mitigation scenarios across world regions. Focusing on scenario variables related to energy and meat consumption, we found a diversity of patterns of justice across scenario characteristics. The prioritarian perspective, which prioritizes improvements to those currently worse off, emerged as the most dominant pattern of justice. By contrast, futures with limited or reduced energy and meat consumption were the least represented in the database. Our research further indicates that most scenarios consistent with patterns of justice do not explicitly aim to model more just futures, suggesting that underlying scenario narratives—most often SSP2—largely determine the distributional outcomes. We therefore propose a stakeholder engagement strategy to make distributional justice assumptions in scenario development ex-ante more diverse and transparent. Overall, this study provides a practical avenue for developing justice-conscious scenarios that may be more likely to motivate collective climate action.

## Introduction

If climate change mitigation scenarios are perceived as unfair or unjust, they are less likely to generate the collective action and support needed to implement them^1–4^. However, developing scenarios that systematically and transparently explore different justice considerations remains challenging and it is largely unknown how different justice-related perspectives shape scenario outcomes.

Developing justice-conscious scenarios is challenging because a clear conceptual link between philosophical concepts of justice and scenario development is missing. Concepts of justice differ fundamentally in their assumptions, origins, and applications. Philosophical justice principles are often elusive to public and political debates, and science communities approach justice from different angles^5^. The proliferation of justice concepts, as well as the quickly growing justice-related literature, can lead to contradictions: some scientific studies on effort-sharing between nation states were found to include allocation principles that are unrelated to normative equity principles^6^. This conceptual fragmentation is further evidenced by a recent systematic review documenting no less than 2167 different indicators for energy, climate, and environmental justice published in academic studies over a 25-year period^7^.

These conceptual inconsistencies extend beyond academic discourse into international policy discussions. For example, nationally determined contributions (NDCs) under the United Nations Framework Convention on Climate Change (UNFCCC) were found to be based on moral justifications disconnected from justice principles recognized by international law^8^, although nation states often state equity considerations to justify their NDCs^9^. Unsurprisingly, nation states have been found to cherry-pick justice principles that are most favorable for themselves^10^.

While justice is a multi-dimensional concept, climate change mitigation policy is often most directly concerned with distributional justice, a collection of theories about a just or equitable allocation of burdens and benefits^2,11,12^. Consequently, scenario studies informing climate policy place emphasis on distributional justice considerations^13,14^. We define a pattern of distributional justice as a distributive principle rooted in philosophical theory that describes a just allocation of some given outcome metric or currency of justice.

Patterns of justice as defined here apply to any metric related to collective wellbeing and are not conceptually bound to mitigation-related considerations. They are therefore broader than fair share principles discussed in the context of the United Nations Framework Convention on Climate Change (UNFCCC), which represent different rationales on how to distribute the global carbon budget or mitigation burdens across countries or country groups^8^^,^^15^. Although these fair share principles (e.g., based on responsibility, capacity, equality, and right to development) can be partly mapped onto different philosophical theories^16^, they have been criticized for not representing a comprehensive perspective on climate or distributional justice and mainly apply to states and not to other actors^17,18^.

The objective of this study is to provide a conceptual framework for analyzing patterns of distributional justice in climate change mitigation scenarios. The main research question is: “To what extent can different patterns of justice be used to systematically and transparently explore distributional justice considerations in climate change mitigation scenarios?”

This main research question is further broken down into two sub-research questions:

SRQ.1) How can patterns of distributive justice be operationalized in the context of climate change mitigation scenarios?

SRQ.2) What patterns of distributive justice are prevalent in current climate change mitigation scenarios, and how do these patterns vary across global temperature outcomes, mitigation strategies, models, Shared-Socioeconomic-Pathways (SSPs) and scenario studies?

To answer these sub-research questions, this study proposes a new conceptual framework to operationalize patterns of justice in the context of climate change mitigation scenarios. In a second step, we apply this conceptual framework to evaluate distributional justice implications of scenarios in the IPCC’s Sixth Assessment Report (AR6) scenario database^19^. The study concludes with a discussion of the applicability of the conceptual framework and reflections about the integration of other justice dimension into the modeling of climate change mitigation scenarios, particularly procedural justice.

By providing a flexible and transparent framework for understanding distributional justice in climate change mitigation scenarios, this study enables a more systematic and reflexive scientific exploration of climate change mitigation strategies and their distributional justice implications.

## Results

### Operationalizing patterns of justice in climate change mitigation scenarios

We propose that philosophical definitions of distributional justice can be meaningfully translated into definitions of just evolutions, and hence trajectory requirements. Here, a trajectory describes the development of a variable over time. Since most quantitative scenarios include temporal simulations of many variables, they consist of many trajectories. Climate change mitigation scenarios produced by integrated assessment models (IAMs), for example, detail carbon dioxide emissions in every modeled timestep, which can be visualized as simple line charts. Variable trajectories can be generated within two different steps of the scenario modeling process. According to Krumm, Süsser and Blechinger^20^ scenario modeling includes (1) narrative development, (2) computational simulation, and (3) discussing scenario outputs.

On the one hand, variable trajectories can be pre-defined by modelers during the first step of scenario modeling as part of the narrative development. The SSP framework, for example, describes different trajectories for population, urbanization, and gross domestic product (GDP)^21^, which subsequently drive energy consumption and land use change in a scenario. In general, scenario narratives often exemplify techno-economic factors rather than underlying cultural, social, political, or equity considerations^22–24^. On the other hand, variable trajectories can be generated within the computational simulation itself. In this case, the variable trajectories depend, amongst others, on the model’s solution algorithm and economic framework. It has been found that the solution algorithms of models used for climate change mitigation scenarios are often based on a utilitarian perspective—they generate scenarios that maximize the sum of utilities or minimize cost^25^. However, the extent to which solution algorithms and economic frameworks affect the resulting variable trajectories is unclear.

Many variables in global climate change mitigation scenarios are reported for different geographical regions, resulting in trajectory sets in which each trajectory represents one region. We propose that analyzing those sets of regional trajectories enables a better understanding of the regional distributional implications of scenarios. However, the conceptual framework presented here is also applicable to other spatial scales.

Our patterns of justice framework is only meaningful for scenario variables that represent a form of utility or disutility. By “utility” we mean objective and measurable factors relevant to collective well-being. For clarity, this paper only refers to variables directly proportional to utility—cases where a variable increase can be understood as an analogy to an increase in well-being. Variables directly proportional to utility are, for example, health benefits, income, or access to energy services. In the case of variables inversely proportional to utility, the interpretations provided below should be inverted.

Following Zimm et al.^14^, there are at least five patterns of distributional justice, which are sometimes referred to as distributional justice “principles” or “shapes”^2^. For brevity and readability, we use the term “patterns of justice” for the remainder of this study when we refer to patterns of distributional justice. We consider five patterns of justice in this study: (1) total utilitarian, (2) prioritarian, (3) egalitarian, (4) sufficientarian, and (5) limitarian. These patterns of justice hold different core claims to what defines a just allocation.

**Fig. 1:**
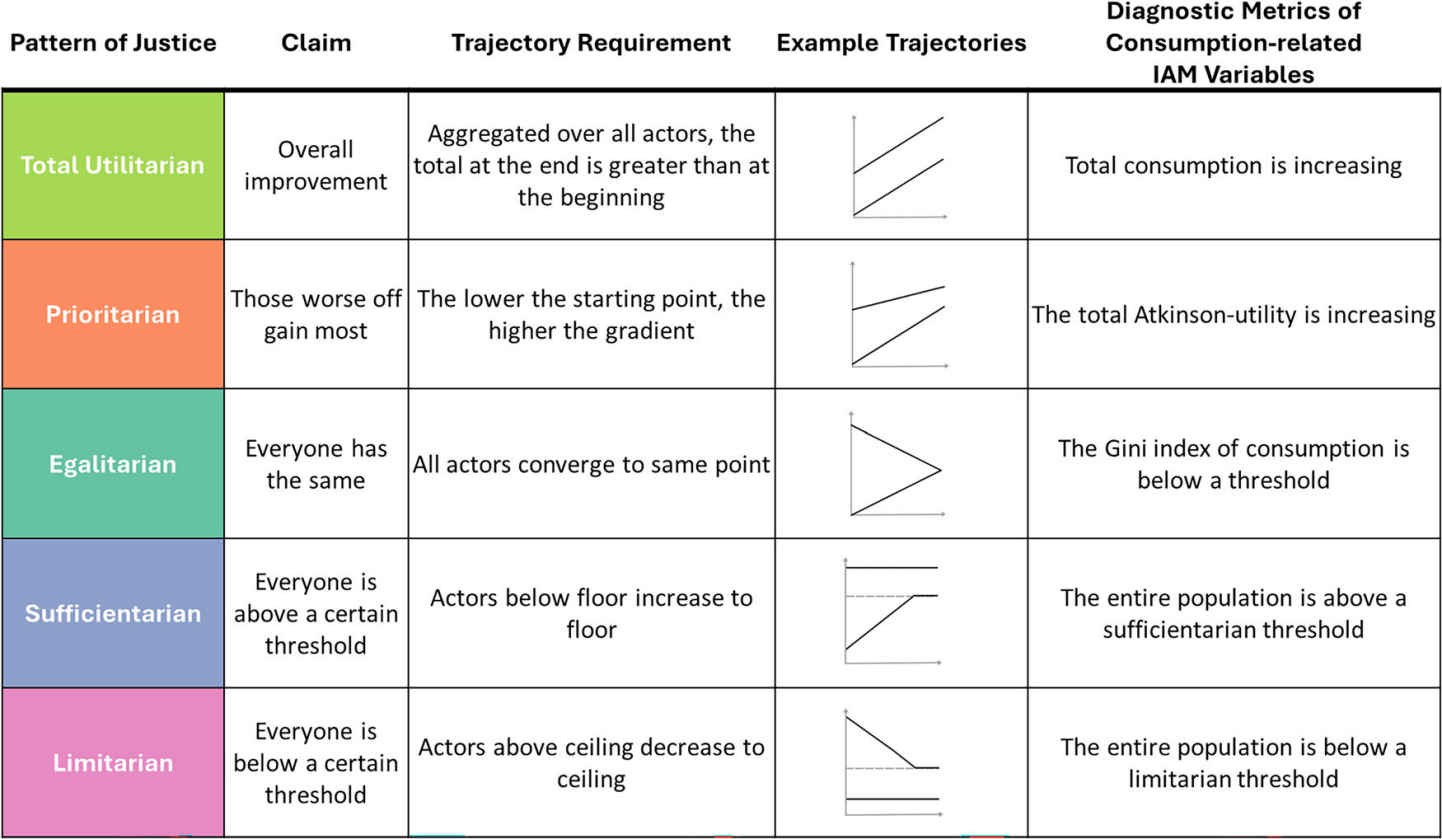
Trajectory requirements consistent with patterns of justice. Five patterns of justice (Total Utilitarian, Prioritarian, Egalitarian, Sufficientarian, and Limitarian) are summarized, including their core normative claims, the trajectory requirements each pattern implies, illustrative example trajectories, and corresponding diagnostic metrics of consumption-related IAM variables.

Figure 1 shows how patterns of justice can be translated into trajectory requirements. Total utilitarianism holds that a just distribution increases the total utility across all actors. In trajectory terms, this implies that the final total across all trajectories must exceed the initial one. Prioritarianism emphasizes the priority of improvements for those worst off over comparable improvements to those better off. This can be represented in trajectories by a steeper gradient of trajectories that represent actors who initially score lower on the variable of interest. The egalitarian pattern requires the elimination of inequalities among all actors, or reducing inequality below a target level. Trajectories can reflect inequality reductions through convergence. The sufficientarian pattern requires every actor at minimum to achieve a defined threshold, while the limitarian pattern requires each to remain below it. This can be translated into the requirement for trajectories to achieve and remain above or below a certain threshold.

It is important to note that patterns of justice as well as trajectory requirements are not mutually exclusive. On the contrary, it may be possible to create scenario trajectories that fulfill requirements of all patterns of justice simultaneously. Indeed, as we show later, we find that trajectories can often be seen as satisfying multiple patterns at the same time.

The “Example Trajectories” column in Fig. 1 presents simplified stylized trajectories that could satisfy the respective justice claims. In all examples, time is shown on the *x*-axis and the variable of interest on the y-axis. The black lines represent the variable value of two illustrative actors, and the gray dotted line marks exemplary thresholds for sufficientarian and limitarian distributions. The last column in Fig. 1 presents diagnostic metrics that can be used to test a trajectory set for consistency with patterns of justice.

In other words, the diagnostic metrics are the last step in operationalizing patterns of justice by numerically describing trajectory requirements. We do not propose an exhaustive set of valid diagnostics; rather, we acknowledge that multiple diagnostics may appropriately capture each pattern. The diagnostic metrics proposed in Fig. 1 are tailored towards evaluating consumption-related variable trajectories in climate change mitigation scenarios generated by IAMs. More precise mathematical definitions of the diagnostic metrics are provided in the next section (see Fig. 8). A scenario whose trajectory set is found consistent with a given pattern of justice can be classified as exploring that pattern in the respective variable. Thereby, the proposed framework enables and requires the evaluation of distributional justice considerations on a variable-to-variable basis.

In summary, we propose to relate patterns of justice to visual “patterns” of variable trajectories. This allows scenarios to be evaluated based on whether their variable trajectories meet one or multiple trajectory requirements. Considering a specific variable, a scenario can be interpreted as partly aligned with a distributional pattern of justice if its variable trajectories meet those requirements. This simple yet effective conceptual link enables scenario modeling to address distributional justice concerns in a transparent and systematic way. The following section demonstrates how this approach supports the evaluation of existing scenarios in the AR6 scenario database. Our aim is to illustrate the application of justice patterns in scenario research and to encourage further work using this or similar methodologies.

### Exploring patterns of justice in IPCC AR6 scenarios

To make philosophical justice theories legible for quantitative climate change mitigation scenarios, we translated patterns of distributional justice into requirements for variable trajectories. These requirements were subsequently expressed mathematically through diagnostic metrics (see Fig. 8) which we applied to evaluate the distributional justice implications of scenarios in the IPCC’s Sixth Assessment Report (AR6) scenario database^19^. It is important to note that the evaluation focuses on whether resulting scenario trajectories are consistent with patterns of justice. As we will show later, this does not imply that the scenarios were specifically designed ex-ante to model just futures or global redistribution.

Because this study focuses on the evolution of trajectories across world regions, only scenarios providing data for at least two R10 regions were considered. Scenarios were further classified into three mitigation strategies, and patterns of justice were assessed across three consumption-related variables under two levels of parameterization (see Methods). The study was also restricted to climate change mitigation scenarios, excluding those outside the IPCC AR6 C1 to C5 climate categories, which fail to limit peak warming to 2.5 °C throughout the twenty-first century with a likelihood of >50%^26^.

The AR6 database includes 574 scenarios within the climate categories C1 to C5 that report the variables required for at least two R10 regions each. Of these 574 scenarios, 255 (45%) were classified as CDR, 197 (34%) as DEMAND, and 122 (21%) as RENEWABLE. Unsurprisingly, CDR scenarios mostly explore higher temperature outcomes (C4, C5), while DEMAND and RENEWABLE scenarios tend to explore lower temperature outcomes (C1–C3).

**Fig. 2:**
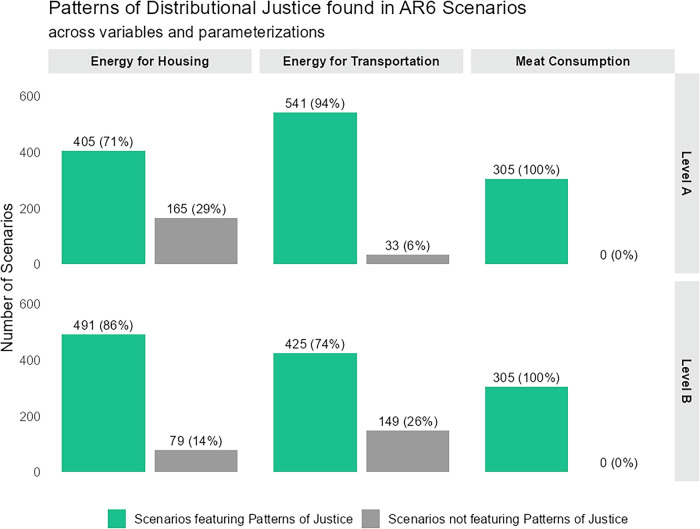
Patterns of distributional justice explored in IPCC AR6 scenarios. Bar charts show the number of IPCC AR6 scenarios featuring (green) or not featuring (gray) patterns of justice across the variables final energy for housing, final energy for transportation, and meat consumption at parameterization levels A and B.

Figure 2 shows that most scenarios include variable trajectories consistent with at least one pattern of justice. When considering energy consumption for housing, 86% of all scenarios were found to relate to at least one pattern of justice under the level B parameterization, and 71% under the more ambitious level A. Considering energy consumption for transportation, more scenarios were found to relate to at least one pattern of justice under the level A (94%) compared to the level B parameterization (74%). This is the result from many scenarios being classified as prioritarian only under the level A parameterization, indicating that many climate change mitigation scenarios assume increases in housing-related energy consumption in currently worse-off regions and decreases in better-off regions, with total energy consumption for housing overall declining. All 305 scenarios that report the variable meat consumption were found to relate to at least one pattern of justice.

**Fig. 3:**
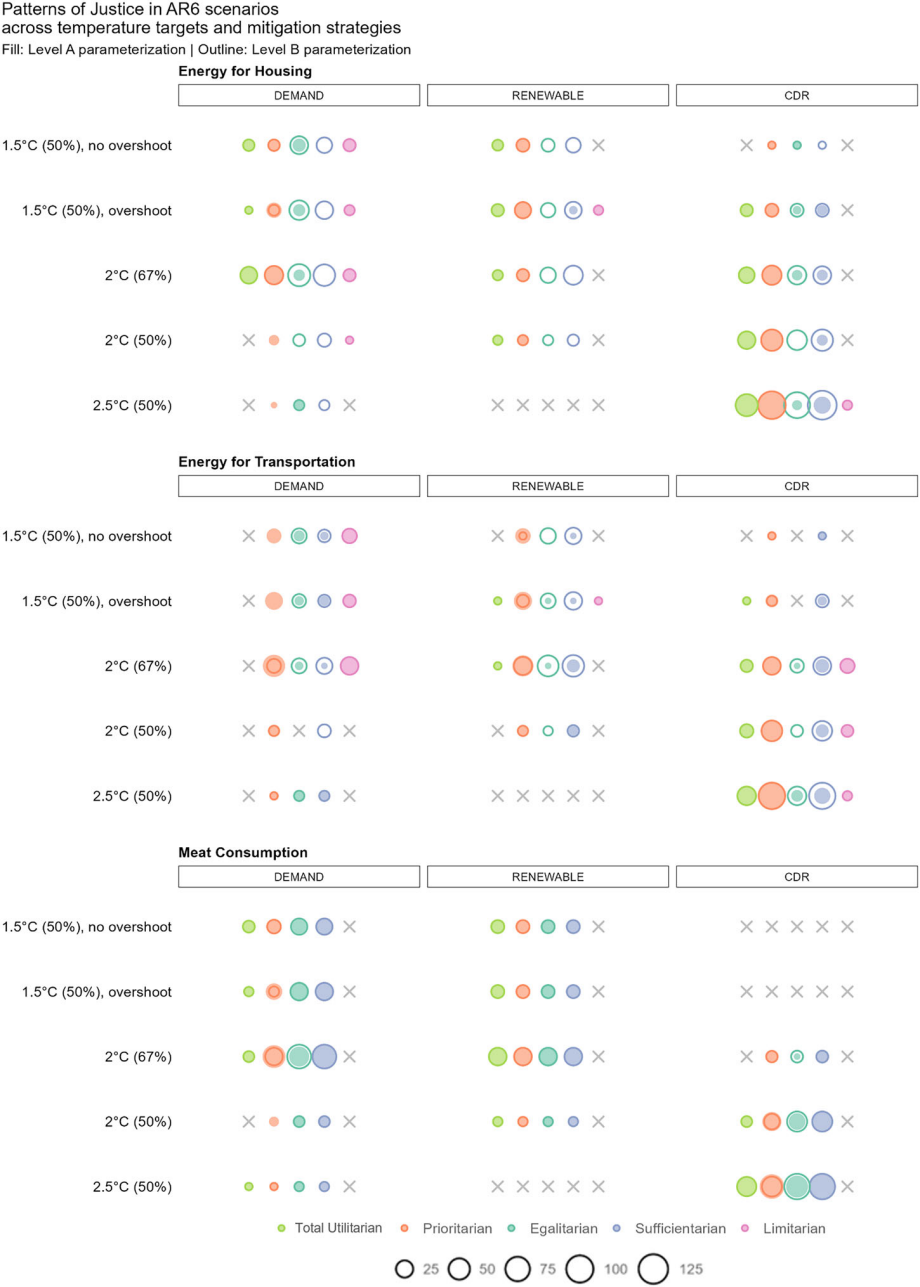
Scenarios in the IPCC AR6 scenario database featuring trajectories consistent with patterns of justice. The bubble color indicates the pattern of justice, and the bubble size indicates the number of scenarios. A gray “X” indicates that no scenario was found to explore the specific pattern of justice in this variable-temperature outcome-mitigation strategy configuration.

Evaluating the occurrence of patterns of justice, Fig. 3 shows a diversity of patterns of justice across all variables, climate categories, and mitigation strategies. Most importantly, we found no indications that patterns of justice are at odds with climate change mitigation: climate categories were not restricting patterns of justice in scenario trajectories. The full diversity of patterns of justice was also observed in the lowest climate category, which avoids overshoot.

The most prevalent pattern of justice in our scenario ensemble was the prioritarian pattern, irrespective of the parameterization. The egalitarian and sufficientarian patterns had greater variation depending on the parameterization, while the total utilitarian and limitarian patterns were, by design, unaffected by the parameterization.

Analyzing patterns of justice across mitigation strategies, we found that CDR scenarios were most consistently reflective of sufficientarian patterns. Large-scale deployment of CDR enables higher total energy consumption—also expressed in the prominence of total utilitarian patterns—which in turn makes it easier to meet sufficiency thresholds. Limitarian patterns in energy-related variables were most consistently attributed to DEMAND scenarios. DEMAND scenarios were also most often consistent with the egalitarian pattern under the more ambitious, level A parameterization. In contrast, RENEWABLE scenarios were almost never consistent with the limitarian pattern.

Egalitarian and sufficientarian patterns were found more frequently than total utilitarian patterns, indicating assumptions about strong increases in energy efficiency. As we show later, most AR6 scenarios assume continuous economic growth consistent with SSP1 or SSP2, which does not necessarily translate into increases in energy consumption.

Further investigating different patterns of justice across variables, we found few scenarios exploring the total utilitarian pattern for *energy consumption for transportation*. Paired with the prevalence of prioritarian patterns, this may indicate a shared paradigm across modeling teams that energy use for transportation will increase only in some regions and not across the entire world. *Meat consumption* was the only variable for which no scenarios were found to follow a limitarian pattern. No scenario featured trajectories consistent with the limitarian threshold, even though this threshold was set slightly above the World Health Organization recommendations. This is surprising because environmental and health implications of meat consumption are well understood, providing clear and reasonable limitarian rationales for meat consumption^27,28^. At the same time, we found a strong and consistent presence of all other patterns of justice in the variable *meat consumption*. For example, across climate categories and mitigation strategies, some scenarios assume increasing meat consumption in all world regions, consistent with the total utilitarian pattern.

To avoid repeatedly presenting results from both level A and level B parameterization, we focus on the outcomes of the more ambitious level A parameterization for the remainder of this section when analyzing the variation of patterns of justice across modeling frameworks, SSPs and scenario modeling projects.

**Fig. 4:**
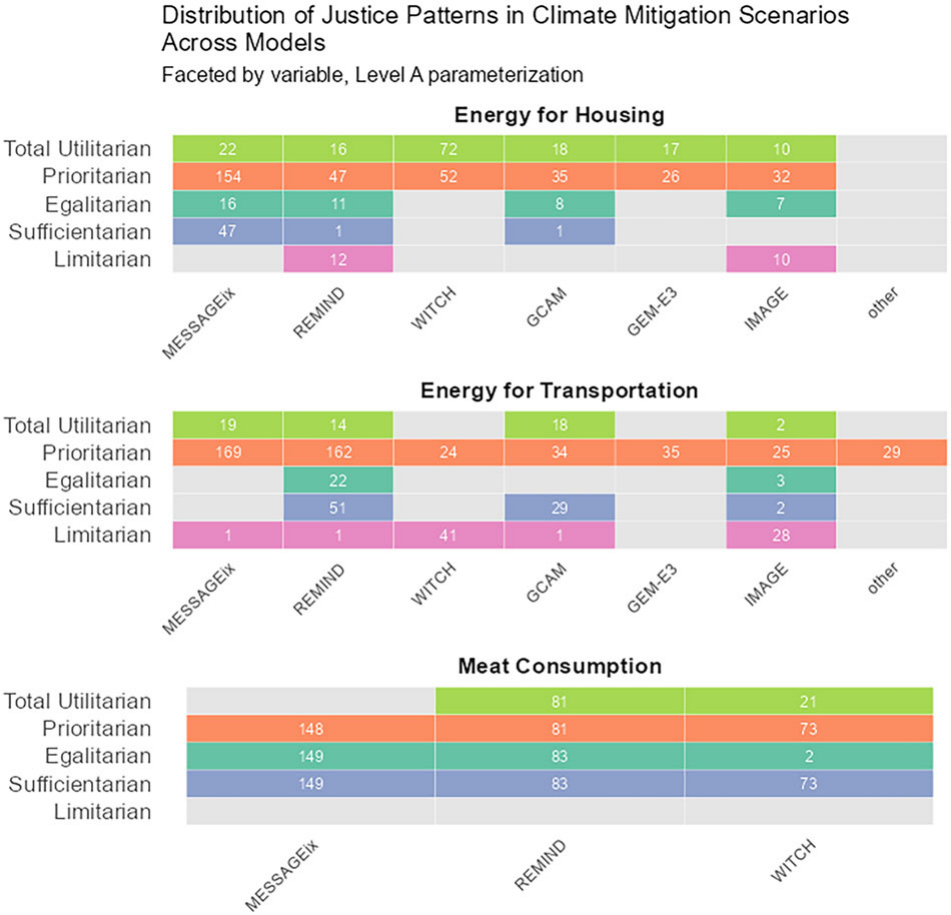
Scenarios consistent with patterns of justice across IAM frameworks. Columns indicate IAM frameworks and rows indicate patterns of justice, shown separately for final energy for housing, final energy for transportation, and meat consumption at level A parameterization. Counts give the number of scenarios; gray cells indicate that no scenario was found for that category.

Analyzing patterns of justice across the six most prominent modeling frameworks in our sample, which account for around 95% of all scenarios, we find no indication that equilibrium type or modeling approach constrain a models’ ability to represent patterns of justice (Fig. 4). MESSAGEix, REMIND, and WITCH are general equilibrium models that apply an intertemporal optimization algorithm, whereas GCAM, GEM-E3 and IMAGE use a recursive dynamic modeling approach^29^. GCAM and IMAGE are further based on a partial equilibrium framework, covering not the entire economy but only certain economic sectors and markets^30^. We also found that, at the time of the publication of the AR6 database and based on the level A parameterization, some modeling frameworks did not yet explore certain patterns of justice in the variables in scope of this analysis. For example, limitarian patterns in *energy for housing* and the egalitarian patterns in *energy for transportation* were found in only two out of the six highlighted modeling frameworks.

**Fig. 5:**
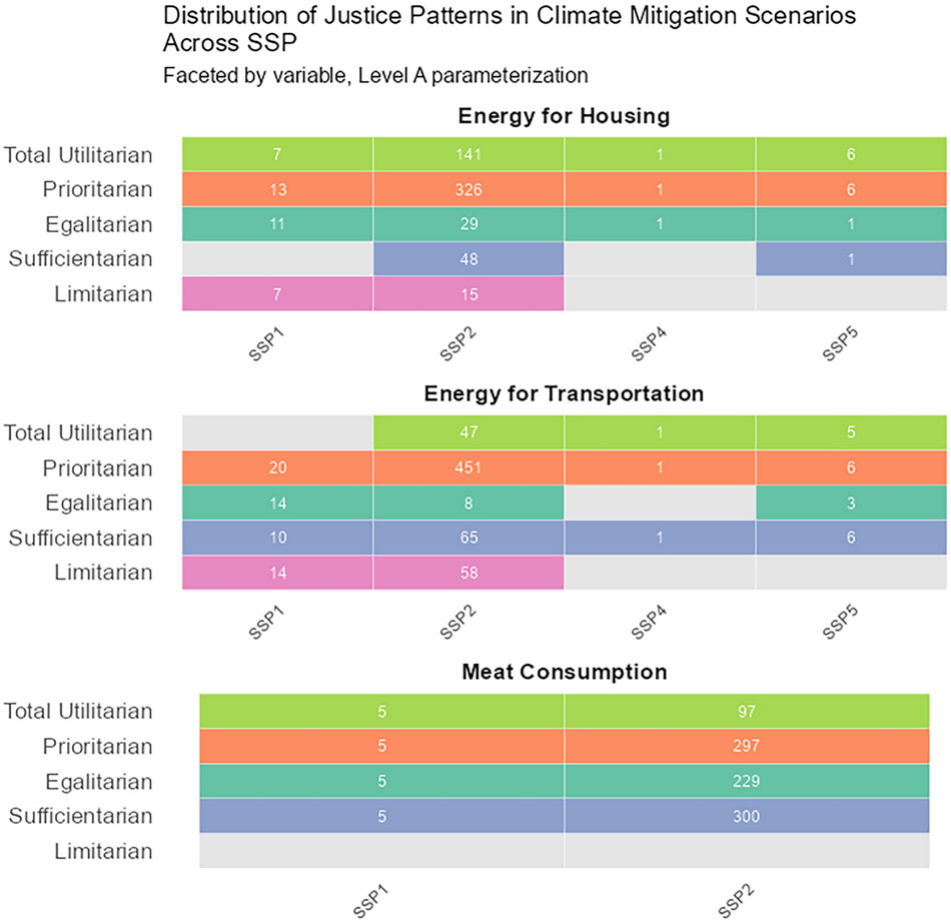
Scenarios consistent with patterns of justice across SSPs. Columns indicate SSPs and rows indicate patterns of justice, shown separately for final energy for housing, final energy for transportation, and meat consumption at level A parameterization. Counts give the number of scenarios; gray cells indicate that no scenario was found for that category.

Justice considerations are an important component of the SSP framework. The SSPs describe global inequalities alongside socio-economic and demographic developments. Analyzing patterns of justice across SSPs assesses the consistency between the SSPs and our patterns of justice framework (Fig. 5).

Starting with SSP1, we surprisingly found the sufficientarian pattern to be unexplored for *energy for housing* in the level A parameterization, which may be at odds with the development-focused and well-being orientation of SSP1. A possible explanation may be that SSP1 scenarios assume large improvements in energy efficiency, reducing energy needs to below thresholds derived from SDP-EI (see Methods). In contrast, it seems plausible that there is no SSP1 scenario exploring a total utilitarian pattern in *energy for transportation*. The combination of SSP2’s economic assumptions—sustained economic growth across the world—and the large number of SSP2-based scenarios available makes it little surprising that we found all patterns of justice explored under SSP2. SSP3, which describes a world of high climate mitigation and adaptation challenges, is not represented in this analysis because there are no SSP3 scenarios in the AR6 database that limit global average temperature to below 2.5 °C. SSP4 is titled “Inequality—a road divided” and describes a world of increasing inequalities. It therefore seems plausible that patterns of justice are largely underexplored in this SSP under the level A parameterization. Similarly, it seems reasonable that SSP5-based scenarios do not explore limitarian patterns. SSP5 assumes rapid economic growth fueled by fossil fuels, which is in conflict with a limitarian perspective.

**Fig. 6:**
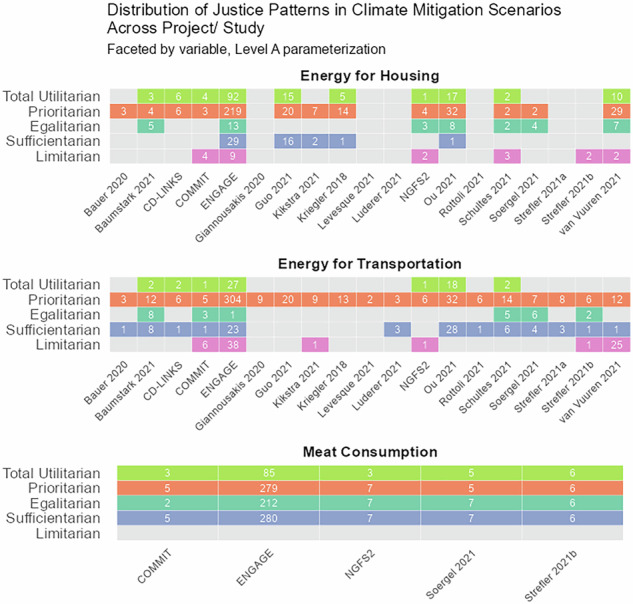
Scenarios consistent with patterns of justice across scenario studies. Columns indicate scenario studies and rows indicate patterns of justice, shown separately for final energy for housing, final energy for transportation, and meat consumption at level A parameterization. Counts give the number of scenarios; gray cells indicate that no scenario was found for that category.

Lastly, we analyzed patterns of justice across scenario studies and found that many studies explored multiple patterns of justice (Fig. 6). Those patterns of justice were often explored in a latent manner—patterns of justice were identified in scenarios developed without an explicit research focus on distributional justice considerations. The international ENGAGE project, for example, had the objective to develop climate mitigation scenarios that meet the objectives of the Paris Agreement while accounting for multidimensional feasibility pathways^31^. Strefler et al.^32^, labeled Strefler 2021b in the AR6 scenario database, researched how carbon removals affect carbon price trajectories, and Ou et al.^33^ explored the interactions between CO_2_ and non-CO_2_ emission trajectories. The terms “justice”, “equity”, and “fairness” are neither explicitly mentioned in ENGAGE’s project description nor in the two research papers, but all developed scenarios that are consistent with diverse patterns of justice in some or multiple outcome variables analyzed. Therefore, we assume that the prevalence of patterns of justice in the AR6 scenario ensemble is largely a consequence of the underlying scenario narratives. As such, our results depend largely on SSP2, which assumes moderate sustainable development and reductions in income inequalities^21^.

**Fig. 7:**
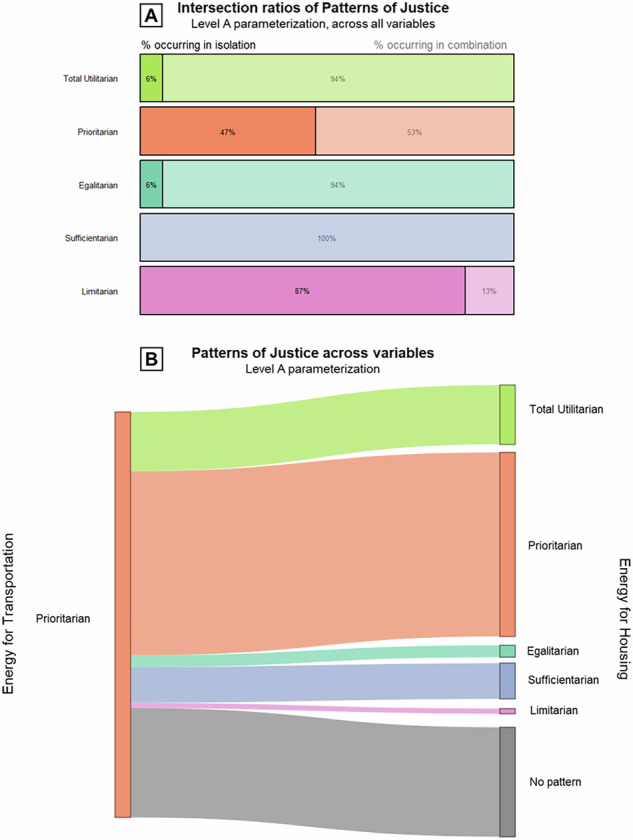
Interactions between patterns of justice. **A** Intersection ratios of patterns of justice at level A parameterization across all variables. Darker segments indicate the percentage of trajectory sets featuring a pattern in isolation; lighter segments indicate the percentage occurring in combination with other patterns. **B** Sankey diagram of scenarios consistent with the prioritarian pattern in final energy for transportation and the patterns they are simultaneously consistent with in final energy for housing, at level A parameterization.

To better understand how the proposed diagnostic metrics attribute patterns of justice to scenarios, it is important to understand the interactions between patterns of justice. How do patterns of justice relate to each other? Shown in Fig. 7A, we found that patterns of justice tend to occur in combinations. Total utilitarian, egalitarian, and sufficientarian patterns occur in combination with other patterns more than 90% of the time. The prioritarian pattern, which on its own occurs the most often in total, still occurs in combination with other patterns more than 50% of the time. The limitarian pattern is an exception because it occurs individually 87% of the time. This is surprising because there is no conceptual argument against merging limitarian patterns with other patterns of justice. The most common combinations are prioritarian-egalitarian-sufficientarian, total utilitarian-prioritarian, and prioritarian-sufficientarian.

We also found that expressing a pattern of justice in one variable does not necessarily imply that the scenario is also consistent with that pattern of justice in other variable trajectories. Figure 7B compares the patterns of 270 scenarios that are exclusively consistent with a prioritarian pattern in the variable *energy for transportation* against the patterns those scenarios are consistent with in the variable *energy for housing*. While a large proportion of scenarios are consistent with a prioritarian pattern in both variables, many scenarios are also consistent with additional patterns. Most importantly, a significant share of scenarios was found not to be consistent with any pattern in the variable *energy for housing* at all, reinforcing the conclusion that distributional implications of scenarios can only be drawn on a variable-to-variable basis.

To summarize, using diagnostic metrics, we identified diverse patterns of justice in AR6 scenario trajectories. Patterns of justice were found across many dimensions: mitigation strategies, temperature outcomes, modeling frameworks, and scenario studies. We did not find any indication that those dimensions restrict the possibility to model patterns of justice in climate mitigation scenarios. The number of scenarios per SSPs in the AR6 dataset is too limited to draw conclusions on how SSPs influence patterns of justice. In other words, we do not find any evidence that certain mitigation strategies or temperature outcomes are incompatible with any pattern of justice, at least with respect to the variables analyzed. Every pattern of justice is explored at least once for every climate mitigation strategy, albeit not for every temperature outcome. The reasonable exemption is the total utilitarian pattern in the variable *energy for transportation* in DEMAND scenarios. This diversity of patterns suggests that it is possible to model a range of just futures using IAMs. The pressing question then becomes which futures to model. We believe that this decision should be made jointly with stakeholders (see Box 1: Stakeholder-informed narratives).

Box 1 Stakeholder-informed narratives With our study showing no clear constraints for patterns of justice in climate change mitigation scenarios, the pressing question becomes which patterns to model. Given limited resources, prioritization is crucial to explore the most relevant patterns of justice for the most salient variables. Involving stakeholders in this prioritization process can address recent calls to open up scenario development^43^ and can enhance the credibility and policy relevance of scenario research. Motivated by Klinsky and Winkler^12^, we call for the patterns of justice framework to be used to co-create scenario narratives that explicitly reflect the evolution of distributional principles over time.

We suggest a simple two-step co-creation process for scenario narratives, based on translating philosophical justice principles into visual trajectories. These visual trajectories are then used to facilitate the elicitation of distributional preferences from stakeholders and the public, who can thus express their preferences in a format that is legible for modeling teams. First, stakeholders identify which scenario variables are most important to them. Second, stakeholders are presented with scenarios containing different trajectories of those variables, with each scenario serving as a stylized representation of a particular pattern of justice. Stakeholders then select the scenario they perceive as the fairest. Eventually, the modeled scenarios can then feature the fairest perceived trajectories for the most important variables.

Such trajectory-based stakeholder engagement can be supported by various tools. To illustrate this, we provide an open-source web application demonstrating how preferences for patterns of justice can be elicited. Users are presented with five scenarios, each displaying fictional trajectories of regional energy service access that correspond to patterns of justice. Stakeholders are then asked to indicate which scenario they perceive to be the fairest (see Fig. 9 for an example). The web app can be adapted to different contexts, and similar applications can be constructed with minimal resources.

## Discussion

This study operationalized five patterns of distributional justice in the context of global climate change mitigation scenarios. We demonstrated that translating patterns of justice into trajectory requirements allows users of our framework to systematically evaluate distributional consequences of scenarios. In this section, we reflect on our findings from assessing the AR6 scenario database and discuss alternative trajectory requirements.

We found no indication that certain patterns of justice are incompatible with certain mitigation strategies. Based on the energy and meat consumption variables analyzed in this study, we suggest that all mitigation strategies are, in principle, compatible with all justice patterns. The sole exception is the combination of a mitigation strategy centered on reducing energy demand and adopting total utilitarianism. Relying primarily on demand reduction for climate mitigation while simultaneously increasing total energy or meat consumption is inconsistent. This is particularly evident in energy use for transportation and meat consumption, where assuming efficiency gains large enough to offset increased consumption seems implausible. However, we observed total utilitarian patterns in meat consumption of scenarios classified as DEMAND scenarios, because the DEMAND classification is based on a scenario’s reported primary energy consumption rather than its meat consumption profiles.

Scenario studies explicitly dedicated to exploring multiple combinations of mitigation strategies and justice patterns may yield highly policy-relevant insights. Justice patterns reflect moral values^34^, and policy positions are often justified on moral grounds. It is therefore critical for decision-makers to understand whether a given mitigation strategy precludes morally desirable futures. In other words, pursuing certain mitigation strategies may render specific justice-related outcomes unattainable.

Similarly, justice-related scenario outcomes appear to be currently largely determined by shared assumptions about future socio-economic developments and, crucially, the SSP framework. Economic activity is a key driver of energy demand in climate change scenarios, and most scenarios base their assumptions about global economic trends on the SSPs^30^. The distributional outcomes observed in this study can be, at least partly, attributed to the prominence of SSP2 narrative in the AR6 scenario dataset. This means that patterns of justice in scenario outcomes are strongly shaped by decisions made in the scenario design, at the beginning of the scenario development process, that do not necessarily target distributive justice considerations.

Future scenario research on distributional justice could explicitly model a range of mitigation strategy–justice principle combinations for multiple SSPs. Scenario studies that limit themselves to a single justice principle, mitigation strategy, or SSP cannot reveal whether fundamental incompatibilities exist between these dimensions. Justice-centered versions of the SSPs could consider providing trajectories that conceptually link directly to patterns of justice, potentially providing a powerful tool to mainstream distributional justice considerations in global climate change mitigation scenarios. For example, Wood et al.^35^ and Kikstra et al.^36^ provide promising starting points for alternative socio-economic assumptions that may encourage stronger egalitarian and limitarian scenario outcomes. In the meantime, to help modelers identify patterns of justice that deserve prioritization, we propose and test a participatory methodology in a text box amended to this manuscript (see Box 1: Stakeholder-informed narratives and Box 2: Pilot stakeholder elicitation).

Building on this study, future conceptual work could explicitly describe patterns of injustice. What are the trajectory patterns that fundamentally contradict patterns of justice? For example, scenarios that assume that the situation of those currently better-off improves while the situation of those worse-off worsens. Similarly, “no-change” scenarios simulating a perpetuation of a status quo in which sufficientarian and limitarian thresholds are not met may prove incompatible with any justice pattern.

The presented findings are dependent on decisions made regarding trajectory requirements, variables, and justice patterns. As indicated earlier, multiple trajectory requirements may be consistent with a particular pattern of justice. Context determines the most suitable trajectory requirement and the thresholds used for assessment. An alternative diagnostic metric for the egalitarian pattern, for example, would be the inter-regional standard deviation. A shrinking standard deviation would reliably reflect decreasing inequalities between regions. However, the standard deviation is not frequently used to measure inequalities, and it is unclear what standard deviations would be consistent with ambitious inequality reductions. For this reason, we relied on Gini indices consistent with income poverty eradication. The Atkinson function, used here to define the prioritarian trajectory requirement, could be replaced by other metrics that measure how the consumption share of those worse off changes over time. For example, a scenario could be classified as prioritarian if the 50% worst-off increase their share of total consumption over time.

After experimenting with similar alternative metrics, we found that metrics tracking consumption shares can lead to incorrect results in scenarios that simulate decreasing consumption across all regions. In such cases, the alternative metrics were found to categorize a scenario as prioritarian if those worse-off decreased less than those better-off, misrepresenting the prioritarian core claim that those worse-off should improve their situation in an absolute, not relative manner. No matter the exact parameterization, the assumed trajectory requirements—and related thresholds—should always be communicated transparently, allowing others to easily understand whether the respective parameterization is consistent with their own interpretation of the patterns of justice.

The binary classification conducted in this study could alternatively be replaced with a graduated scale. It could be argued that scenarios explore patterns of justice to varying extents, for example, if most but not all trajectories of a scenario pass a sufficientarian threshold. Nevertheless, we deliberately decided against a graduated scale as the value of the resultant descriptive findings on proportions of scenario trajectories would be unclear.

This study focused on final energy and meat consumption, representing a narrow subset of material variables under the broader justice metric of “well-being” and reflecting limitations to comprehensive justice assessments due to a limited number of widely reported variables across the AR6 database. No assumption is made that our variable selection captures the only appropriate variables, just that they are indicative, relevant, and empirically tractable for our purposes. Future research could explore the distribution of other justice-relevant metrics, such as power^37,38^ or health, in scenarios. The assumed future location of energy infrastructure or CDR technologies, for example, has profound implications for geopolitical competition.

Moreover, analyzing alternative food-related variables beyond meat consumption would be beneficial to better understand the nutritional implications of scenarios. However, such analysis would require models to consistently report additional variables, such as population at risk of hunger^39^. More fundamentally, further social science research is needed to identify which justice-related metrics are most salient to people. In the realm of housing, for example, does the distribution of the energy used for heating, floor space, or access to green spaces evoke stronger distributional concerns amongst members of the public? At the same time, distributional concerns can be expected to vary depending on spatial scale, with global and local inequalities being perceived differently.

Many of the trajectories analyzed in this study arise from optimization algorithms that operate on a utilitarian least-cost paradigm and that are calibrated on a current, highly unjust global economy. Earlier research described alternative solving algorithms that are more likely to result in just outcomes^25^. While those algorithms may be preferable for justice-focused modeling exercises, we found no indication that current solving algorithms pose a barrier to simulating patterns of distributional justice. As changing the solving algorithms of modeling frameworks is often understood as a daunting, resource-intensive task, it may be an attractive alternative to enforce variable trajectories in scenarios by pre-specifying them in the stage of narrative development.

Next to distributional justice, there are other forms of justice that are yet to be incorporated into scenario design^14^. For example, there are intensifying calls to open up quantitative scenario development to other scientific fields and non-scientists^40,41^, as well as to use very different research designs such as citizen science, participatory action research, indigenous methodologies, feminist methodologies, and experimental ethnography, among others^42^. More inclusive scenario development would advance the procedural justice of scenarios^43^, which relates to the design of decision-making procedures^14^. In Box 1 appending this manuscript, we present an idea to facilitate broader public engagement in scenario development by representing questions of distributional justice through visual trajectories.

To conclude, this study proposes a new conceptual framework to transparently and systematically evaluate patterns of distributional justice in climate change mitigation scenarios. First, philosophical patterns of distributive justice are translated into trajectory requirements. Second, diagnostic metrics are used to test the trajectory sets of scenarios for consistency with those trajectory requirements. We found variable trajectories to be consistent with diverse patterns of justice across mitigation strategies, temperature outcomes, modeling frameworks, and scenario studies. Prioritarianism emerged as the dominant pattern in the AR6 scenario dataset, and limitarianism was the least represented. Given that no hard constraints for patterns of justice were detected, we conclude that patterns of justice are mostly dependent on assumptions made at the stage of narrative development. Accordingly, SSP2 is considered a major driver of the patterns of justice identified in the AR6 dataset. As we show in the accompanying boxes, the presented patterns of the justice framework are not only useful to evaluate existing scenarios, but they can also be a powerful tool for co-creating stakeholder-informed narratives about just allocations.

Box 2 Pilot stakeholder elicitation In a pilot field experiment involving 24 respondents, we found that our visual-driven survey supports stakeholders in expressing and reflecting on their preferences, and that the web-app facilitated group discussions about distributional justice. In the pilot study, the egalitarian pattern was the most preferred pattern. However, when asking participants about why they preferred a certain pattern, we found that sufficientarian and limitarian motivations (“Everyone should be above a certain threshold”, “There should be a limit for consumption”) were more widespread than the egalitarian motivation (“Everyone should have the same consumption level”). Our pilot study also confirmed prior research showing that perceptions of fairness depend on the metric allocated^60^. Users expressed different preferences for patterns of justice depending on the energy service considered, and preferences varied with the thresholds applied in sufficientarian, limitarian, and egalitarian trajectories. It seems reasonable to expect that a different selection of regions or countries would also influence preferences.

The pilot illustrates how the patterns of justice framework can facilitate stakeholder engagement in scenario narrative development. At the same time, observing how their preferences change across different contexts can provide valuable and surprising insights for participating stakeholders. We believe that systematic stakeholder engagement based on visualized trajectories can be a powerful tool for participatory scenario development, providing valuable understanding about which trajectories are the most relevant to model. This can enhance scenario credibility, not only by accounting for distributional justice but by promoting procedural justice. By discussing preferred trajectories, stakeholders who are often excluded from modeling processes can be actively involved. They can contribute contextual knowledge, help identify relevant variables, shape the construction of diagnostic metrics, and offer insights into the desirability of selected patterns of justice. We believe that applying the proposed methodology marks a critical step towards co-creating socially legitimate and, indeed, more procedurally just scenarios.

## Methods

To address this study’s second sub-research question, the prevalence of patterns of justice across global temperature outcomes, mitigation strategies, models, Shared-Socioeconomic-Pathways (SSPs), and scenario studies, we first assign scenarios to generalized mitigation strategies. We then apply mathematical formulations of the proposed scenario diagnostics to evaluate the conformity of IAM scenario trajectories with patterns of justice.

Our analysis of patterns of distributional justice in the context of global climate mitigation scenarios from IAMs focuses on the scenarios included in the scenario database of IPCC’s Sixth Assessment Report (AR6)^19^.

This study is restricted to world regions using the R10 regional aggregation, including: Eastern Asia (primarily China), South Asian countries (primarily India), other Asian countries, Pacific OECD countries, Middle Eastern countries, Sub-Saharan African countries, the Reforming Economies of Eastern Europe and the Former Soviet Union (primarily Russia), Eastern and Western Europe (i.e., the EU28), Latin America and the Caribbean, and North America.

**Fig. 8:**
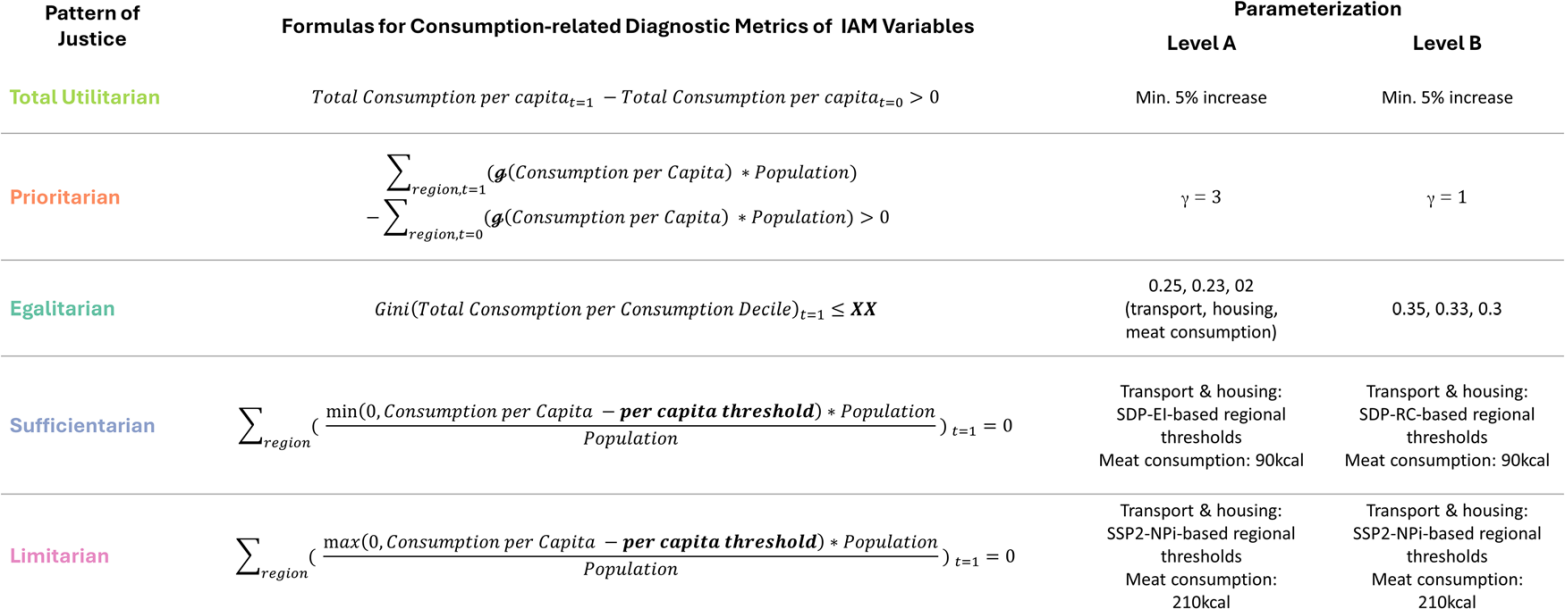
Diagnostic formulas and parameterizations. Parameters defined on the right are highlighted in bold in the formulas. ${\mathcal{g}}$ refers to the Atkinson function. In this study, *t* = 0 refers to 2020 and *t* = 1 refers to 2050.

The analysis aims to achieve global coverage, so that patterns of justice of scenarios can be linked to their global temperature outcomes and cover a large proportion of the available scenario literature. To this end, patterns of justice are assessed on a R10 scale since most scenarios in the AR6 scenario dataset do not report more disaggregated results. While this technical choice limits the examination of the intra- and inter-country distributions, which are also relevant and more tangible^44^, it does facilitate the analysis of global inequality trends for a representative portion of the global climate change mitigation scenario literature. To ensure comparability across regions with different population sizes, we apply population weighting where appropriate (see Fig. 8). As large intra-regional inequalities in energy consumption exist^45^, conclusions at the level of regional sub-populations or individuals fall outside the scope of this analysis.

**Fig. 9:**
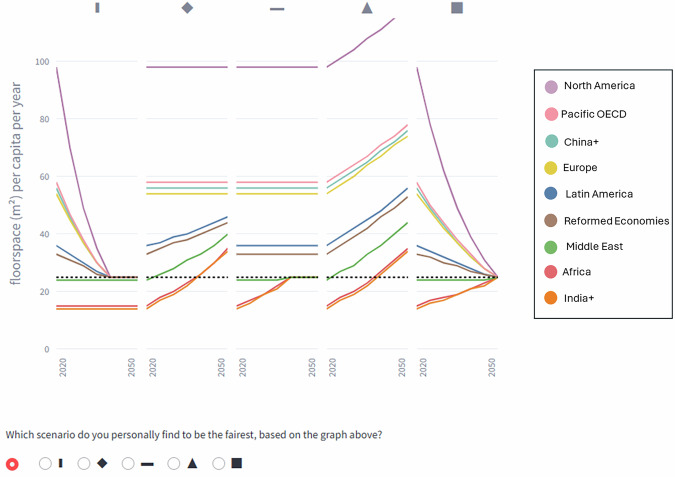
Screenshot of the web application. The screenshot shows regional developments in floorspace per capita per year across five scenarios. 2020 values reflect empirical data. Each scenario is a stylized representation of a pattern of justice. The corresponding patterns of justice are limitarian, prioritarian, sufficientarian, total utilitarian, and egalitarian. The dashed line indicates the threshold used for the limitarian, sufficientarian, and egalitarian patterns and is described in more detail on the web app. In this example, the dashed line symbolizes a floorspace of 25 m^2^.

### Assigning scenarios to mitigation strategies

The mitigation strategies considered are: (1) reducing global aggregate primary energy demand (abbreviated as DEMAND), (2) large-scale deployment of carbon dioxide removal technologies (CDR), and (3) expanding deployment of renewable energy technologies (RENEWABLE). These strategies follow the scenario classifications used in the illustrative mitigation pathways (IMPs) in the AR6^26^ and the first *State of CDR Report*^46^. While effective climate mitigation will require a combination of all available strategies, climate change mitigation scenarios rely on these strategies to varying degrees.

Scenarios were assigned to mitigation strategies using the following procedure. First, scenarios in the AR6 database were grouped by their climate category, which reflects the expected global temperature outcome by 2100 corresponding to a scenario’s projected emissions. Within each climate category, scenarios were then assigned to mitigation strategies using simple heuristics that focused on the primary energy profiles of the scenarios.

The following heuristics were used to assign scenarios to mitigation strategies. Scenarios exhibiting a reduction in total primary energy demand at a global level by 2050 relative to 2020 were classified as DEMAND scenarios. CDR scenarios were characterized by a global increase in total primary energy demand over the same period and by featuring more total primary fossil energy production than total primary renewable energy production by 2050. Since all scenarios were climate mitigation scenarios, this combination—rising energy demand and extended fossil fuel use—implies a stronger reliance on CDR. On the other hand, scenarios that assumed increasing total energy demand by 2050 but featured more total primary renewable energy than from fossil fuels by 2050 on a global level were classified as RENEWABLE scenarios.

The variables required for this classification are primary energy production (*“Primary Energy”* in the IAMC nomenclature*)*, primary energy produced with fossil fuels *(“Primary Energy|Fossil”)*, and primary energy produced through renewable energy technologies *(“Primary Energy|Non-Biomass Renewables”* and *“Primary Energy|Biomass”)*. Scenarios that did not report those variables were excluded from the analysis. The proposed heuristics are simple, transparent, and mutually exclusive, and were deliberately formulated inclusively to maximize the number of models and scenarios in our sample. The resulting scenario categorization is robust to small changes in the heuristics (Supplementary Material, Fig. SM.1).

### Testing scenario trajectories for consistency with patterns of justice

After having assigned scenarios to mitigation strategies, we applied the diagnostics listed in Fig. 8 to evaluate regional scenario trajectories across three consumption-related variables: energy consumption in the transport sector (IAMC format: “*Final Energy|Transport”*), energy consumption in the residential and commercial buildings sector (“*Final Energy| Residential and Commercial”*), and meat consumption (‘*Food Demand|Livestock”*).

There are multiple motivations for selecting these variables. First, these variables represent energy consumption—and associated emissions—connected to services for well-being. They also relate to major household emission sources. Mobility, housing, and the food sector were found to largely determine household carbon footprints^47–49^. The food sector is represented through the variable meat consumption because production of meat is the sector’s largest driver of emissions^50^, and at the same time, very unequally distributed^51^. Lastly, the variable selection was also motivated by the wide availability of these variables in the AR6 dataset.

Other variables, such as energy services, could provide a much more suitable proxy for well-being, but are seldom available in the AR6 scenario dataset. This study acts as a proof of concept of the proposed conceptual framework, and similar analyses could be conducted for other variables, depending on the intended use or policy interest.

A key assumption underpinning the calculation of diagnostics is the period during which scenario trajectories are evaluated. Both the classification of scenarios into mitigation strategies as well as the evaluation of patterns of justice focus on the time period between 2020 (*t* = 0) and 2050 (*t* = 1), as we believe it is more relevant to the reader to learn about the near-term justice implications of scenarios.

To attribute scenario trajectories to patterns of justice, we propose diagnostic metrics, which are displayed in Fig. 8. For each pattern of justice, multiple alternative diagnostics could have been applied. The presented diagnostics are selected because they effectively capture the core claim of each pattern of justice and because they are simple, ensuring an accessible and transparent analysis. To assess the sensitivity of our results to the diagnostics’ parameterization, we apply two different parameterizations for the prioritarian, egalitarian, and sufficientarian patterns of justice: level A and level B, with level A being considered a more ambitious interpretation of patterns of justice than level B. The parameterization does not vary for the total utilitarian and limitarian patterns because we are more confident in the validity of the proposed parameters. For the same reason, the sufficientarian and limitarian thresholds for meat consumption remain the same across parameterizations.

We propose a scenario is labeled “total utilitarian” for a certain variable if the total per capita consumption increases between 2020 and 2050. To account for negligible small increases, totals must increase by at least 5%. Further, we propose a scenario is labeled “prioritarian” when the total, regionally-weighted Atkinson utility increases over time. The Atkinson utility is calculated via the Atkinson function, which is a concave aggregating function giving greater weight to improvements of those worse-off^25,52^. The Atkinson function is defined as ${{\mathcal{g}}}_{y}\left(u\right)={\sum }_{i=1}^{N}{h}_{\gamma }\left({u}_{i}\right)\,where\,{h}_{\gamma }\left({u}_{i}\right)=\left\{{\mathrm{ln}}\,{u}_{i}\,for\,\gamma =1\,or\,{(1-\gamma )}^{-1}{u}_{i}^{1-\gamma }\,for\,\gamma \,\ne \,1,\,\gamma > 0\right\}$, where *u* is the utility profile (vector of utilities of concerned actors) and *γ* is the inequality aversion parameter^25^. According to Żebrowski et al.^25^, “*the parameter γ determines the tolerable degree of losses involved in utility transfers from better-off to worse-off*” (p.18). *γ* equals 1 means that the utility loss from redistribution cannot exceed the utility transferred, whereas *γ* equals 3 means that considerable total utility loss is acceptable^52^. In their sensitivity analysis of the social cost of carbon, Adler et al.^52^ explore *γ* values ranging from 0 to 3.

The “egalitarian”, “sufficientarian”, and “limitarian” diagnostics require setting thresholds. In the case of the egalitarian pattern, a threshold is required as a reference value signaling meaningful improvements. In contrast, the sufficientarian and limitarian patterns are conceptually defined in relation to specific thresholds. Setting valid sufficientarian and limitarian thresholds for future energy consumption is particularly challenging. This is because future energy demand is expected to differ significantly from today’s level, with substantial uncertainties surrounding future energy efficiency gains, technological developments, lifestyle changes, and transformations of provisioning systems. The two parameterizations reflect varying assumptions about future regional energy needs. Both parametrizations build on the assumptions made as part of the Sustainable Development Pathways (SDPs), a set of scenarios developed after the release of the AR6 scenario database that explore synergies between sustainable development and climate action^53^. Kikstra et al.^54^ use the post-processing model DESIRE to calculate region-specific energy requirements to eliminate energy deprivation along the SDPs. We use the SDP scenario with the highest energy demand, called *Economy-driven Innovation* (EI), to motivate the level A parameterization. In SPD-EI, the amount of energy required to eliminate energy deprivation is higher than in other SDP scenarios, leading to a higher sufficientarian threshold. Consistent with this logic, the level B parameterizations of sufficientarian energy consumption thresholds are based on the SDP with the lowest final energy demand, named *Resilient Communities* (RC). More information on the SDP scenarios is provided in the scenario database of the SHAPE project^53^. The regional limitarian thresholds for energy consumption remain the same across both parameterizations and are based on the SSP2-NPi scenario used by Kikstra et al.^54^. The SSP2-NPi scenario represents a future that continues current economic and population trends without additional climate policies. The minimum requirement for a limitarian energy consumption trajectory, hence, is defined as a trajectory that features lower levels of regional energy consumption than a scenario without significant climate action.

Assumptions regarding sufficientarian and limitarian thresholds for meat consumption are directly grounded in medical recommendations. We therefore regard those thresholds as more robust, and they are identical across both parameterizations. The sufficientarian threshold for meat consumption is set to 90 kcal per person per day, based on the Lancet Healthy report^55^, which is in line with the latest report of the EAT-Lancet Commission^28^. Following the reasoning of the report, we do not set the sufficientarian threshold to 0 kcal because we do not want to prescribe abstinence from meat consumption. The limitarian threshold for meat consumption is set to 210 kcal, which is approximately the highest meat consumption recommended by the British National Health Service^56^, using the rule of thumb that 100 g of red meat has around 230 kcal^57^. This threshold is a conservative estimate, to reflect large current inequalities in meat consumption across regions^51^ and little anticipation of decreasing meat consumption in high-income countries in FAO’s pathways to 2050^58^. For comparison, the proposed threshold of 210 kcal exceeds the recommendation of the World Health Organization, which recommends between 98 g–500 g of red and processed per week per adult^59^, equaling about 32 kcal to 163 kcal per day using the same rule of thumb.

No scenario in the AR6 database features absolute convergence in energy or meat consumption across all world regions by 2050. Consistent with our approach for the sufficientarian and limitarian patterns, we rely on Gini index estimates from the SDP-EI scenario to derive an ambitious threshold for the strict egalitarian diagnostic. SDP-EI assumes very strong inequality reductions consistent with income poverty eradication. Accounting for within-country inequality, SDP-EI features global Gini indices of energy consumption of 0.25 for transportation and 0.23 for housing. Current global inequalities in energy consumption for housing and transport are 0.45 and 0.75, respectively, when including within-country inequality^54^. We set the strict egalitarian Gini threshold for meat consumption to 0.2 and increase all Gini indices by 0.1 points for the level B parameterization.

Applying the described diagnostics on the AR6 dataset, we make as few adjustments to the original data as possible. Population scaling is always based on the scenarios’ own population reporting, and we do not correct for missing data. For each variable, scenarios not reporting the required data are excluded from the analysis. While the proposed trajectory requirements, expressed through diagnostic metrics, are based on literature, they are, of course, not conclusive. The merit of the patterns of justice framework is not to provide definite diagnostic metrics or thresholds, but providing flexible framework that responds to ongoing calls for greater transparency in value-based decisions and clearer connections to philosophical principles^2,12,15^.

## Supplementary information

Supplementary Information

## Data Availability

All datasets and documents used for the analyses in this paper are derived from publicly available sources. These data sources are specified in the “Methods” text and in the analysis source code.

## Code availability

The underlying code for this study is available on GitHub and can be accessed via this link: https://github.com/zyankarli/patterns_of_distributional_justice. More information on the web application and its source code can be found via this link: https://github.com/zyankarli/INCLISA.
