# Supplementary material for: Exploring patterns of distributional justice in global climate change mitigation scenarios
Source: NPJ Clim Action. 2026 Mar 31;5(1):39. doi: 10.1038/s44168-026-00364-4 (PMC13035473; doi:10.1038/s44168-026-00364-4)
Supplement: Supplementary file 1 — Supplementary Information [file 44168_2026_364_MOESM1_ESM.pdf]

Supplementary material

Robustness of heuristics

We use heuristics to assign climate change mitigation scenarios in the AR6 dataset to mitigation strategies. The heuristics are based on two thresholds.

First, scenarios that report a reduction of total primary energy demand in 2050 compared to 2020 are assigned to the DEMAND category. Figure SM.1 shows that most C1 to C5 scenarios assessed report demand changes between -75 EJ and 100 EJ, indicating that scenarios in the DEMAND category assume only moderate demand reductions over the considered time period. Heuristics that would require a stronger signal of decreasing energy demand would label fewer scenarios as belonging to the DEMAND category.

Secondly, scenarios with increasing primary energy demand are differentiated based on their primary energy profile in 2050. Scenarios that report more primary energy from renewables than from fossil fuels are assigned to the RENEWABLE category. The CDR category holds scenarios that report more primary energy from fossil fuels than renewables in 2050.

The difference between the majority of scenarios in the RENEWABLE and CDR categories is substantial, with a separation of at least 150 EJ between the lower RENEWABLE and upper CDR quartile. This difference is considerable given that total primary energy demand across most scenarios ranges between 400 and 800 EJ in 2050. This indicates that most scenarios in the two categories are widely separated, even though a minority of scenarios fall closer together.

To conclude, the applied heuristics are robust to small changes in cut-off values, but larger adjustments would lead to markedly different categorization, and, hence, different results.

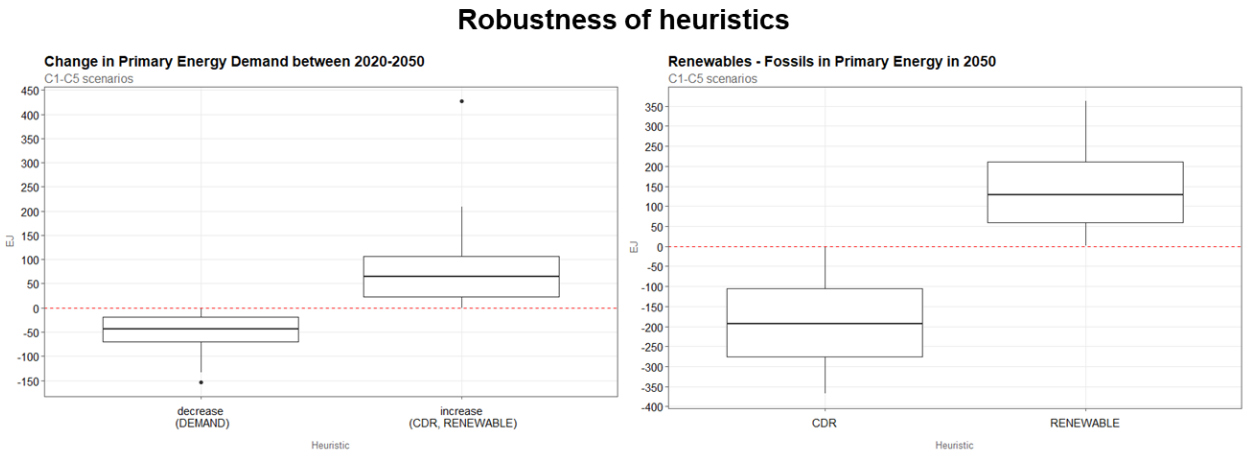

***Figure SM. 1**: Robustness of heuristics. Left: Boxplots of changes in primary energy demand between 2020 and 2050 for scenarios that are categorized as DEMAND, CDR, and RENEWABLE. Right: Boxplots of primary energy profiles for scenarios categorized as CDR and RENEWABLE. The y-axis shows the difference between primary energy from renewables and primary energy from fossil fuels for each scenario.*
